# Fiberoptic endoscopic evaluation of swallowing (FEES) in children with spinal muscular atrophy type 1: feasibility, swallowing safety and efficacy, and dysphagia phenotype

**DOI:** 10.1007/s00405-024-08922-4

**Published:** 2024-09-04

**Authors:** Francesco Mozzanica, Nicole Pizzorni, Marco Gitto, Claudia Dosi, Anna Mandelli, Sofia Gandolfi, Alessandro Campari, Riccardo Masson, Antonio Schindler

**Affiliations:** 1 Department of Clinical Sciences and Community Health, Università Degli Studi di Milano, Milan, Italy; 2 Department of Otorhinolaryngology, IRCCS Multimedica, Milan, Italy; 3 Department of Biomedical and Clinical Sciences, Università degli Studi di Milano, Via GB Grassi 74, Milan, 20157 Italy; 4 Division of Pediatric Anesthesia and Intensive Care Unit, Department of Pediatrics, Children’s Hospital Vittore Buzzi, Milan, 20154 Italy; 5 Developmental Neurology Unit, Fondazione IRCCS Istituto Neurologico Carlo Besta, Milan, Italy; 6 Department of Paediatric Radiology, Children’s Hospital Vittore Buzzi, Milan, Italy

**Keywords:** Spinal muscle atrophy (SMA), Fiberoptic endoscopic evaluation of swallowing (FEES), Videofluoroscopic swallow study (VFSS), Dysphagia phenotype, Swallowing safety, Swallowing efficacy

## Abstract

**Purpose:**

Although dysphagia is a common symptom among patients with Spinal Muscular Atrophy Type 1 (SMA1), scant data exist on the application of Fiberoptic Endoscopic Evaluation of Swallowing (FEES) in this population. The aim was to analyze FEES feasibility, swallow safety and efficacy, dysphagia phenotype, and agreement with VideoFluoroscopic Swallow Study (VFSS) in children with symptomatic, medication-treated SMA1 and oral feeding.

**Methods:**

10 children with SMA1 underwent FEES. Six patients had also a VFSS. Two clinicians independently rated FEES and VFSS videos. Swallowing safety was assessed using the Penetration-Aspiration scale (PAS). Dysphagia phenotypes were defined according to the classification defined by Warnecke et al. Swallowing efficacy was evaluated with the Yale Pharyngeal Residue Severity Rating Scale (YPRSRS) in FEES, whereas pharyngeal residue was rated as present or absent in VFSS.

**Results:**

FEES was performed in all children without complications. Four children tolerated bolus trials during FEES, in 4 children swallowing characteristics were inferred based on post-swallow residues, while 2 children refused to eat and only saliva management was assessed. The dysphagia phenotype of predominance of residue in the piriform sinuses was documented in 7/8 children. The PAS score was < 3 in 3 children and > 5 in one child. Swallowing efficacy was impaired in 8/8 children. VFSS showed complete agreement with FEES.

**Conclusions:**

FEES is a feasible examination in children with SMA1. Swallowing safety and efficacy are impaired in nearly all patients with strong agreement between FEES and VFSS. Dysphagia is characterized by the predominance of residue in the piriform sinus.

## Introduction

Spinal Muscular Atrophy Type 1 (SMA) 1 is a rare autosomal recessive neuromuscular disorder characterized by progressive muscle weakness and atrophy. It affects approximately 1:11.000 live births [1] and is caused by a mutation in the Survival Motor Neuron 1 (SMN1) gene on chromosome 5 which leads to a deficiency of the survival motor neuron (SMN) protein [2]. Type 1 is the most common and severe form of SMA, with onset before 6 months of age. Affected infants typically display progressive muscle weakness, poor muscle tone, and difficulty moving and they never achieve the sitting position. If left untreated, SMA1 frequently leads to death within the first 2 years of life because of a progressive impairment of motor functions, including respiratory muscles [3]. Dysphagia is also a common symptom among patients with SMA1 and is related to the weakness of the muscles involved in chewing and swallowing [2, 4]. For this reason, dysphagia complications including aspiration pneumonia, malnutrition, dehydration, and poor growth, are frequently documented in affected patients [2, 5].

Until recently, despite the dramatic impact of the above-mentioned factors on disease progression, dysphagia has been managed only with palliative approaches [2]. A major change arose thanks to the introduction of new treatment modalities which proved to be effective in increasing the life expectancy and motor skills of affected patients. Currently, there are three approved therapies for SMA: nusinersen, onasemnogene abeparvovec, risdiplam. Nusinersen is administered by intrathecal injection and acts as a splicing modifier of the SMN2 gene. Onasemnogene abeparvovec is a single-dose intravenous viral vector carrying a copy of the SMN1 gene. Risdiplam is orally administered daily and modifies the SMN2 pre-messenger RNA splicing [6]. Despite the significant improvement in motor functions, the effect of these treatments on swallowing abilities is not entirely clear. For example, Weststrate et al. [7] did not demonstrate a significant improvement nor preservation of swallowing functions in patients treated with nusinersen. On the contrary, Van der Heul et al. [8] found that children treated with nusinersen showed an improvement or preservation of their swallowing functions which, however, deteriorated again after 5 months.

In addition, to the best of our knowledge, only three studies [6, 9] focused on the analysis of swallowing characteristics of treated patients with SMA1 using validated scales and instrumental examinations such as Fiberoptic Endoscopic Evaluation of Swallowing (FEES) or Videofluoroscopic Swallow Study (VFSS), which represent the two gold standards in dysphagia diagnostics [2]. Two studies used the VFSS. Chacko and colleagues [9] characterized swallowing function in children with SMA treated with disease-modifying agents, including 8 patients with SMA1 aged from 6 months to 8 years who underwent VFSS, while Choi et al. [10] recruited 11 patients with SMA1 aged from 3 to 11 years. Only Zang et al. [6] described swallowing function in 10 children with SMA1 based on FEES examinations. In particular, FEES has several advantages compared to VFSS: it is feasible in different clinical settings, doesn’t require radiation exposure, allows testing different types of food, and enables the assessment of the swallowing anatomy and the effectiveness of compensatory strategies [11]). However, the authors of this study [6] reported data on secretions management, but data on bolus management were not clearly described and no information on the main mechanism of dysphagia (dysphagia phenotype) was reported. Consequently, very little is known about: (1) the underlying physiological correlates responsible for dysphagia in these patients; (2) the severity of swallowing safety impairment (defined as the ability to adequately protect the airway from entry of ingested material [12]); and (3) the severity of swallowing efficacy impairment (defined as the ability to transport food and liquids from the oral cavity and through to the esophagus without post-swallow residue [12]). Finally, also the feasibility of VFSS and FEES in the assessment of swallowing abilities in SMA1 patients has been poorly studied.

Considering that the newly available treatments increase the survival of patients with SMA1, the problem of how to guarantee the most suitable safe nutrition becomes crucial. In other words, it is essential to understand “if” and “what” SMA1 patients could orally eat, avoiding pulmonary and nutritional complications.

This study aimed to gather more information regarding FEES in the assessment of dysphagia in patients with SMA1. In particular, we are reporting data concerning: (i) the feasibility of FEES, (ii) swallow safety and efficacy, and (iii) dysphagia phenotype in children with symptomatic, medication-treated SMA1 and oral feeding. Additionally, the secondary aim was to explore the agreement on dysphagia phenotype between FEES and VFSS in those children who underwent both instrumental assessments of swallowing.

## Materials and methods

This study is a retrospective case-series. The study was conducted following the Declaration of Helsinki. Ethical approval was not sought for the present study because of the retrospective design and all data were gathered as part of routine hospital care for which written consensus is collected for each patient or caregiver.

### Participants

Ten children with confirmed SMA1 and treated with one of the three currently approved therapies were assessed using FEES at the dysphagia center of our hospital between January 2021 and February 2023 to estimate the risk of swallowing-related complications. The inclusion criteria were: partial or full oral feeding and a negative history of pulmonary complications. Exclusion criteria were: pulmonary infection or other temporary medical condition that could interfere with swallowing at the moment of FEES procedure.

**Fig. 1:**
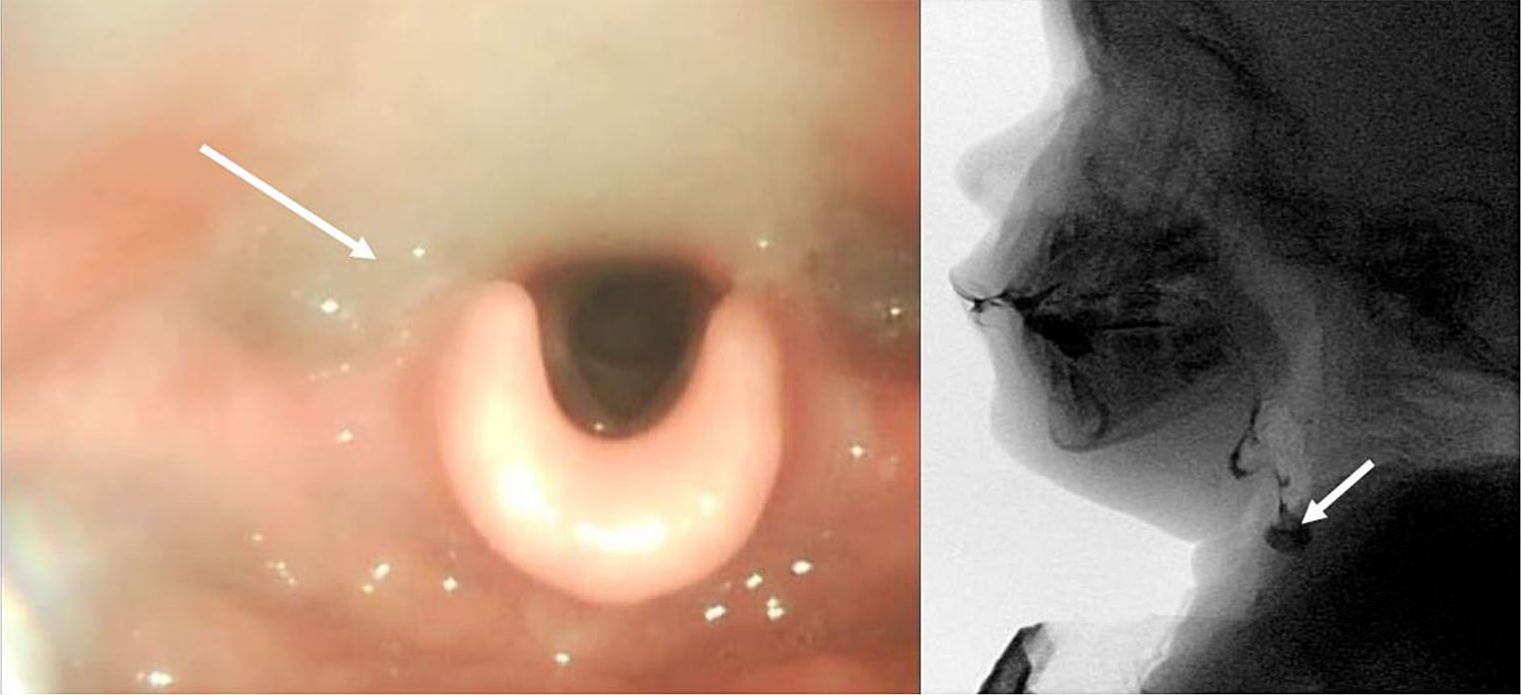
FEES and VFSS example of residue. Picture of FEES and VFSS examination of patient 10 are reported. In both examinations residues in the pyriform sinus (white arrows) are visible

The pediatric version of the Functional Oral Intake Scale (p-FOIS) [13] was used to collect information regarding patient’s oral intake. It is a five-point ordinal scale indicating limitations in oral feeding which ranges from 1 (nothing by mouth) to 5 (total oral diet without special preparation or compensation). The FOIS was administered immediately before the FEES examination based on parents’ reports of daily diet. Parents were also asked about the duration of an average meal at home. Body weight and height were measured for each patient before the swallowing assessment. All the parents received exhaustive explanations regarding the aims and objectives of the FEES evaluation and all the possible risks involved.

### FEES examination

Each FEES was conducted by a senior Phoniatrician with the aid of a Speech and Language Therapist (SLT) and a nurse. A high-definition flexible videoendoscope with a diameter of 2.9 mm (Storz Cmos 11102CM, Tuttlingen, Germany) was used. All the videos were stored in an anonymous form in AVI format.

All the children were examined in the position usually applied at home. No local anesthetic drugs (e.g., lidocaine spray) were used in order not to alter pharyngo-laryngeal sensibility [11]. The endoscope was introduced into the widest nasal cavity and kept at a level just inferior to the uvula to maximize the field of view, including the larynx, the glossoepiglottic valleculae, and the pyriform sinuses [14]. The analyzed textures were age- and development-appropriate and were administered via the tools that the children use at home (bottle, spoon, syringe).

Before the FEES examination, the parents were asked about the different consistencies of their child’s everyday meals. Since we assumed low compliance for the FEES examination, the consistency with the highest level on the International Dysphagia Diet Standardisation Initiative (IDDSI) framework [15] that represented the habitual feeding of the patient was used for the examination. Before FEES, the same consistency was first provided by the parents in the outpatient’s room to appraise possible swallowing impairment signs such as choking. According to the IDDSI framework [15], 3 possible consistencies were tested:

- Mildly thick liquids (IDDSI level 2): room temperature thickened water;
- Pureed food (IDDSI level 4): room temperature Crème Line vanilla pudding (Nutrisens Medical SAS, Francheville, France) (2583.3 ± 10.41 mPa·s at 50s-1 and 697.87 ± 7.84 mPa·s at 300s-1; IDDSI Level 4);
- Dissolvable hard solids (IDDSI level 7): a quarter of shortbread cookie.

FEES videorecordings were prospectively and independently rated by two clinicians, a Phoniatrician and an SLT with over 10 years of experience in dysphagia assessment, management, and FEES ratings, using validated ordinal scales for swallowing safety and efficacy [11]. Videos were presented in random order. Raters were blinded to each other and participants’ data since videos were stored in an anonymous form. Disagreements were resolved by consensus.

Different parameters were analyzed using the FEES examination:

- Dysphagia phenotypes were defined according to the FEES classification defined by Warnecke et al. [16]: (1) premature bolus spillage, (2) delayed swallowing reflex, (3) predominance of residue in the valleculae, (4) predominance of residue in the pyriform sinus, (5) pharyngolaryngeal movement disorder, (6) fatigable swallowing weakness, and (7) complex disorder. The characteristic pattern of FEES identified as the main mechanism of dysphagia was considered the main phenotype [16].
- Safety impairment (penetration/aspiration): the safety of swallowing was rated using the Penetration-Aspiration Scale (PAS) [17]. The PAS is an 8-point scale ranging from 1 (materials do not enter the airway) to 8 (materials enter the airway, pass below the vocal folds and no effort is made to eject). Penetration was defined as the bolus entering the laryngeal vestibule over the rim of the larynx (PAS score from 2 to 5). Aspiration was defined as the bolus passing below the true vocal folds (PAS score 6 or above). Based on the PAS score, each swallow was classified as unsafe if the material entered the laryngeal vestibule (PAS ≥ 3) [11, 18]. Besides quantifying safety impairment, raters examined whether penetration/aspiration occurred before, during, or after pharyngeal swallow; the whiteout period during FEES examination was considered the marker of pharyngeal swallow.
- Efficacy impairment (pharyngeal residue): the amount of pharyngeal residue after the swallow in the valleculae and the pyriform sinus was rated using the Yale Pharyngeal Residue Severity Rating Scale (YPRSRS) [19–21]. The YPRSRS is a 5-point scale ranging from 1 (no residue) to 5 (severe residue). For each level, a percentage, an operational definition, and an anchor image are provided. A YPRSRS score ≥ 3 (mild residue) was considered suggestive of an ineffective swallow [11].

### Videofluoroscopic swallow study

A VFSS was performed when FEES examination was not sufficient to decide whether to continue oral feeding. VFSS was performed by an experienced pediatric radiologist and technologist with a Luminos digital fluoroscopy system (Siemens Healthcare). Patients were seated in a highchair or lying in a crib mounted on the videofluoroscopic table, examined in the lateral projection while consuming barium contrast (Prontobario HD, Bracco Imaging, Italy) mixed in water (IDDSI 1) and/or semisolid food (yogurt or fruit smoothie, IDDSI 4). Digital fluoroscopy images were acquired at 15 or 30 pulses per second. The standard field of view included the oral cavity and the pharynx, using lips, cervical spine, nasal cavity, and upper esophageal segment as reference points. To allow for the evaluation of fatigue impact while minimizing radiation exposure, fluoroscopy was turned on for visualization of 5 sequential swallows at 4 standard time points (min: sec): 00:00, 00:30, 01:30, 02:30 [22]. Information regarding the penetration/aspiration was gathered using the PAS scale. Residue after swallow was rated as present or absent. Dysphagia phenotypes were defined with the same methods used for FEES [16]. The VFSS examinations were prospectively and independently rated by the same two clinicians who evaluated the FEES videos. Videos were presented in random order. Raters were blinded to each other, to the results of FEES examination, and to participants’ data since videos were stored in an anonymous form. Disagreements were resolved by consensus.

### Statistical analysis

Inter-rater reliability of FEES scoring between the two clinicians was analyzed. Weighted kappa with quadratic weighting was calculated [23] for the parameters: safety, efficacy, and dysphagia phenotypes. K values were judged as follows: ≤0.20 poor agreement, 0.21–0.40 fair agreement, 0.41–0.60 moderate, 0.61–0.80 good, and 0.81–1.00 very good [24]. A k value of 1 was considered a complete agreement. The same test was also used to analyze the agreement between FEES and VFSS for the PAS scores and the dysphagia phenotypes. Statistical significance was taken at *p* < 0.05.

## Results

Characteristics of the recruited population are reported in Table 1. There were 5 males and 5 females with a median age of 27 months (range 9–57 months). Five children were treated with onasemnogene abeparvovec, 3 children with risdiplam, and 2 children with nusinersen. Median BMI was 13.25 (range 12-15.5).

**Table 1:** Characteristics of the enrolled population

Pt	Sex	Therapy	Age at the beginning of treatment (months)	Age at FEES examination (months)	Meal duration (minutes)	Weight (kg)	Height (m)	BMI
1	M	OA	4	9	60	6.8	1.03	14.6
2	F	OA	5	57	15	12	1.39	12.2
3	F	OA	5	11	20	7.6	0.70	15.5
4	F	OA	12	15	20	9.8	1.26	13.3
5	F	OA	14	16	15	10	1.25	13.8
6	M	Risdiplam	17	33	30	9.6	1.27	12.7
7	M	Risdiplam	17	27	30	11.5	1.38	12.0
8	M	Risdiplam	43	50	40	10.8	1.34	12.2
9	M	Nusinersen	2	44	20	11.1	0.90	13.7
10	F	Nusinersen	3	27	60	11.1	1.27	13.2
Median (IQ)			9.5 (4.25–16.5)	27 (13–41.25)	25 (20–37.5)	11 (10.5–11.5)	0.87 (0.85–0.93)	13.25 (12.3–13.8)

*Note* The median and interquartile ranges (IQ) are reported

*Legend* Pt = patient; F = female; M = male; OA = onasemnogene abeparvovec; BMI = body mass index

### Feeding

The results of p-FOIS scores were collected at the time of the FEES examination. Most of the patients were fed by mouth. In particular, 5 out of 10 patients had a FOIS score of 4 (total oral intake requiring special preparation or compensations or not expanded from whole bottle feeding), and 2 had a p-FOIS score of 5 (total oral intake without special preparation or compensations). 3 patients had a tube and oral feeding in parallel (FOIS = 3).

### FEES examination

FEES examination was performed in all the children with SMA1 and no complication occurred. 4 of them well tolerated the exam, and the swallowing characteristics were analyzed during the bolus trials with the fiberendoscope located at a level just inferior to the uvula. Other 4 patients did not tolerate the exam and, consequently, their swallowing characteristics were inferred by evaluating the pharyngeal and laryngeal conditions immediately after the bolus ingestion (the child swallowed a bolus, and immediately after the ingestion the operator inserted the endoscope in the widest nasal cavity). In these cases, a static endoscopic swallow evaluation (SEES) was performed [25]. The remaining 2 patients refused to eat and consequently only saliva management (pooling of secretions) was assessed during the endoscopic evaluation.

Mildly thick liquids (IDDSI 2) were tested in 2 patients, pureed consistency (IDDSI 4) was tested in 4, and dissolvable hard solids in 2 (IDDSI 7). The time required to complete FEES never exceeded 5 min.

#### Dysphagia phenotypes

Data on dysphagia phenotypes are reported in Table 2. All the patients exhibited at least one dysphagia phenotype. The presence of residues either in the valleculae or in the pyriform sinuses were the characteristics more frequently documented (7 out of 8 patients). Four patients showed only one isolated phenotype, and the remaining 4 showed two combined phenotypes (Table 2). It should be noted that in those patients who were unable to tolerate the evaluation of swallowing with the endoscope placed in the pharynx during the swallowing act, phenotypes 1 and 2 (premature bolus spillage and delayed swallowing reflex) could not be assessed.

**Table 2:** Dysphagia phenotypes at Fiberendoscopic Evaluation of Swallowing (FEES) and Videofluoroscpic Swallow Study (VFSS) in the cohort of patients

Pt	Sex	pFOIS	Consistency during FEES	FEES feasibility	Main FEES phenotype	Other FEES phenotype	Main VFSS phenotype	Other VFSS phenotype
1	M	3	/	Saliva management	NP	/	Normal	/
2	F	4.5	IDDSI 7	Bolus trial	Res Pir	/	NP	/
3	F	4	IDDSI 4	Bolus trial	Res Pir	Res Val	NP	/
4	F	5	IDDSI 4	Bolus trial	Prem Spil	/	NP	/
5	F	3	IDDSI 4	SEES	Res Val	Res Pir	Res Val	Res Pir Prem Spil
6	M	3	/	Saliva management	NP	/	Res Pir	/
7	M	4	IDDSI 2	SEES	Res Pir	Res Val	Res Pir	Res Val
8	M	4.5	IDDSI 7	SEES	Res Pir	/	Res Pir	Res Val Prem Spil
9	M	4	IDDSI 2	Bolus trial	Res Pir	/	NP	/
10	F	5	IDDSI 4	SEES	Res Pir	Res Val	Res Pir	Res Val Prem Spil

The results of the pediatric Functional Oral Intake (pFOIS) as well as the dysphagia phenotypes are reported

*LEGEND* Pt = patient; F = female; M = male; IDDSI = International Dysphagia Diet Standardisation Initiative; SEES = Static endoscopic evaluation of swallowing; Prem Spil = premature bolus spillage; Res Pir = predominance of residue in the piriform sinus; Res Val = predominance of residue in the vallecula; Normal = normal swallowing; NP = not performed

#### Safety impairment (penetration/aspiration)

Swallowing was considered safe in 2 out of 8 patients (Table 3). Among patients who were able to tolerate the FEES examination with bolus trials (*n* = 4), aspiration (PAS = 6) was demonstrated in 1 patient, and penetration (PAS = 3) was found in 2 patients; in all 3 patients penetration/aspiration occurred after the swallow. One patient was scored 1 on PAS. Among the 4 patients only assessed with SEES, 3 had penetration, and 1 exhibited no post-swallow signs of lower airway invasions (Table 3). In the patients assessed through SEES, it was not possible to analyze whether penetration/aspiration occurred before, during, or after pharyngeal swallow.

**Table 3:** Safety and efficacy of swallowing at Fiberendoscopic evaluation of swallowing (FEES) in the cohort of patients

Pt	Sex	Consistency	FEES feasibility	PAS	YPRSRS-v	YPRSRS-*P*
1	M	/	Saliva management	/	/	/
2	F	IDDSI 7	Bolus trial	3	3	3
3	F	IDDSI 4	Bolus trial	1	3	3
4	F	IDDSI 4	Bolus trial	6	2	3
5	F	IDDSI 4	SEES	3	5	5
6	M	/	Saliva management	/	/	/
7	M	IDDSI 2	SEES	5	4	5
8	M	IDDSI 7	SEES	2	3	4
9	M	IDDSI 2	Bolus trial	3	3	5
10	F	IDDSI 4	SEES	1	4	4
Median (IQ)				3 (2.25–3)	3 (3–4)	4 (3–5)

The penetration aspiration score (PAS), Yale Pharyngeal Residue Severity rating scale vallecula (YPRSRS-V), and Yale Pharyngeal Residue Severity Rating Scale pyriform sinus ((YPRSRS-P) are reported. The median and interquartile ranges (IQ) of PAS, YPRSRS-V and YPRSRS-P are also reported

*Legend* Pt = patient; F = female; M = male; IDDSI = International Dysphagia Diet Standardisation Initiative; SEES = Static endoscopic evaluation of swallowing

#### Efficacy impairment (pharyngeal residue)

Ineffective swallow was found in all the patients. In particular, the median YPRSRS vallecula score was 3 (interquartile range 3–4), while the YPRSRS pyriform sinus score was 4 (interquartile range 3–5) (Table 3).

The agreement between the two raters in determining the safety, efficacy, and dysphagia phenotypes was complete (k = 1 for all the 3 parameters).

### Videofluoroscopic swallow study

VFSS was performed in 6 patients (the 4 who did not tolerate the exam and the 2 who refused to eat during FEES). Liquids (IDDSI1) were tested in all the patients, while pureed food (IDDSI 4) was tested in 3 patients.

As far as the PAS scores with liquids are concerned, swallowing was considered safe (PAS < 3) in 2 out of 6 patients. Specifically, 4 out of 6 patients had clinically significant penetration (PAS = 3 in two cases; PAS = 4 in one case; and PAS = 5 in one case). In one patient no penetration or aspiration were demonstrated (PAS = 1). Among the 3 patients who were tested also with semisolids, swallowing was considered safe in 2 out of 3 patients, while the remaining patients had penetration (PAS = 3). In the 4 patients who were analyzed with both VFSS and FEES the agreement in detecting penetration was complete (k = 1).

Residue was rated as present in 5 out of 6 patients. Among those who underwent both VFSS and FESS, the agreement between the two examinations in determining the presence of residues was complete (k = 1; an example is provided in Figure 1).

As far as the dysphagia phenotypes are concerned, the presence of at least one dysphagia phenotype was demonstrated in 5 out of 6 patients. The presence of residues either in the valleculae or in the pyriform sinuses was the characteristic more frequently documented (5 out of 5 patients), followed by premature bolus spillage (3 out of 5 patients). The agreement between VFSS and FEES in determining the dysphagia phenotype was complete (k = 1).

## Discussion

In the present study, we reported information regarding the feasibility of FEES, swallowing safety/efficacy, and dysphagia phenotypes in children with symptomatic SMA 1, medication-treated and oral feeding. The dysphagia phenotypes have been analyzed for the first time in this population.

FEES was performed in all the enrolled patients and no complications were documented. The time required to complete the exam never exceeded 5 min and the inter-rater agreement was complete. As far as the feasibility is concerned, a proper evaluation of the swallowing act was possible in 4 out of 10 children. In the remaining cases, the swallowing characteristics could be only inferred in 4 patients (SEES), while in 2 the FEES could not be performed because of food refusal. These findings are difficult to compare since there is only one study that analyzed the feasibility of FEES in patients with SMA1 [6]. Zang et al. [6] were unable to perform the FEES in 2 out of 10 children but concluded that FEES was a feasible and safe instrumental procedure for infants and children with type 1 SMA. In addition, the data on FEES feasibility reported in this study on SMA1 children, are in line with those reported on mixed populations of children with dysphagia, with a FEES or SEES feasibility in almost 70% of the examined population [24]. We, therefore, assume that FEES or SEES are valid options for the assessment of swallowing in SMA1 children, especially when signs of dysphagia are suspected on clinical examination or medical history.

### Dysphagia phenotype

The presence of residues after swallowing, either in the valleculae or the pyriform sinuses, was the dysphagia phenotype more frequently documented. The high prevalence of this swallowing impairment is in line with the findings of Zang et al. [6] who reported post-swallow residues in all except one examination. Dysphagia phenotype is difficult to discuss since in none of the previous studies the main mechanism of dysphagia was defined. However, McGrattan et al. [2] who analyzed the physiological correlates for dysphagia, found that patients with SMA1 were characterized by profound deficits in sucking efficacy, tongue base retraction, pharyngeal stripping wave, hyolaryngeal movement, and upper esophageal opening. All these factors may contribute to inefficient bolus clearance from the pharynx and are coherent with the dysphagia phenotypes described in this study [2]. As SMA1 is due to motor neuron degeneration that ultimately results in weakness and progressive atrophy due to denervation, it is not surprising that the dysphagia phenotype in this population is characterized by inefficient bolus clearance due to tongue base and pharyngeal weakness.

### Safety impairment (penetration/aspiration)

Penetration of ingested foods was quite common and was found in 5 out of 8 patients. This datum agrees with the reports of Zang et al. [6] who reported penetration in approximately half of the cohort. On the other hand, aspiration was detected in only 1 out of 8 patients, while in the study of Zang et al. [6] aspiration was found in 4 patients. Similarly van der Heul et al. [8] and Choi et al. [10] who analyzed the swallowing abilities in children with SMA1 using VFSS reported that 4 out of 5 and 5 out of 6 patients respectively had silent aspiration. These diverging results might be related to the volume and texture of the ingested foods. For example, in the study of Zang et al. [6] even if the authors stated that large volumes were avoided and that the administered boluses were age- and development-appropriate, no information regarding how many consistencies were tested during each FEES, as well as no information about the consistency more frequently associated with aspiration is available. In our study, we tested only the consistency with the higher level on the IDDSI framework which represented the patient’s habitual feeding, and this could represent a reason for the relatively low prevalence of aspiration in our cohort. In addition, it must be noted that even if 4 patients did not tolerate the FEES and consequently their swallowing characteristics were inferred by evaluating the pharyngeal and laryngeal conditions immediately after the bolus ingestion (thus preventing the detection of pre- or per-deglutition silent aspiration), the VFSS performed in these patients failed to detect aspiration.

Of the 4 patients who tolerated FEES, 3 presented safety impairment; in all of them penetration/aspiration occurred after pharyngeal swallow; this datum is in line with the dysphagia phenotype previously described.

### Efficacy impairment (pharyngeal residue)

Pharyngeal residue in the valleculae and pyriform sinus was detected in all the patients regardless of the texture of the ingested food, thus suggesting an impairment in the bolus propelling from the oropharynx to the esophagus. Even in a recent study on SMA1 children, whose swallowing was assessed through VFSS, abnormal pharyngeal residue was the most common finding [9]. The data reported in our study are in line with those of previous studies [2, 6] and are probably related to the inefficient bolus clearance from the pharynx due to poor tongue base retraction, pharyngeal stripping wave, hyolaryngeal movement, and upper esophageal opening.

The secondary aim of the study was to analyze the agreement between FEES and VFSS. The data reported show that the two gold standards in dysphagia assessment provide similar information on swallowing safety, efficacy, and dysphagia phenotype. No other study compared FEES and VFSS in SMA1 children and therefore our data are difficult to compare to previous literature. Nonetheless, in previous studies comparing FEES and VFSS in children with dysphagia, the results were in line with those of the present study [25, 26]. While this information is clinically relevant, it must be taken with great caution as the two examinations are difficult to compare as they were performed on different days and settings and used different consistencies.

Although the data of the present study should be considered with caution due to the restricted number of recruited patients, they bear several clinical implications to be considered. First, FEES and SEES can be easily applied and provide useful information in the majority of children with SMA1. Given the wide clinical range of neurological phenotypes of SMA1 pharmacologically treated children, instrumental assessment should be considered in the case of clinical suspicion of feeding difficulty. In particular, as FEES and SEES abnormal findings are strong predictors of abnormal VFSS findings [24], VFSS might be restricted to patients with normal FEES or SEES or intolerance to FEES, but clinical suspect of swallowing impairment. Second, the main dysphagia phenotype is represented by residue in the pharynx, which is also the main mechanism leading to penetration/aspiration. Dysphagia compensation (diet modification, bolus volume, meal timing) should be based on this datum. Third, FEES and SEES may use food from everyday life; the findings are therefore easier to translate into diet prescription and family counseling. However, it should be considered that FEES (and VFSS) may miss the assessment of swallowing fatigue, which is one of the main features of SMA patients. Furthermore, despite the high feasibility of instrumental evaluations, FEES and VFSS cannot always be easily included in routine clinical practice.

Finally, as FEES is feasible and provides useful information, when possible it can be used as a frontline tool to monitor the evolution of dysphagia in SMA1 children and clinical decision-making, while VFSS can be used as a second option tool for more selected cases.

## Study limitations

There are several limitations in this study. First, the number of enrolled patients is small, even if in line with previous studies. The patients are SMA1-treated children, selected for the suspicion of dysphagia. Analogously to the other available studies, recruited patients were heterogeneous for age and disease modifying treatment. Consequently, the results here reported should be considered with caution. However, due to the rarity of the disease, consideration of each case is precious. In addition, during the FEES examination, only the consistency with the highest score according to the IDDSI framework which represented the children’s habitual feeding was used. Consequently, no information regarding the swallowing abilities with other consistencies is available. Moreover, a proper FEES examination was performed in only 4 out of 10 patients. In the remaining 6, the examination was not performed at all (in 2 patients), or only partially performed (in 4 patients in which the swallowing abilities could be only inferred because the endoscope was introduced only after the swallow). Finally, the dysphagia phenotypes were judged as present or absent according to the classification proposed by Warnecke et al. [16] whose psychometric properties still need to be analyzed.

## Conclusion

Children with SMA1 are frequently affected by swallowing disorders particularly related to inefficient bolus clearance of the pharynx, due to poor tongue base retraction and pharyngeal contraction and conditioning after pharyngeal swallow penetration/aspiration. The FEES examination could be considered a useful tool for swallowing evaluation in selected patients with SMA1 because of its feasibility and good inter-rater agreement. The possibility to test everyday foods may represent a key advantage of FEES over VFSS in this population, by easing the acceptability of food during the examination and providing more ecological information.

## Data Availability

All data were gathered as part of routine hospital care.
